# The Last Straw: How Stress Can Unmask Parkinson’s Disease

**DOI:** 10.3233/JPD-230400

**Published:** 2024-06-04

**Authors:** Anouk van der Heide, Claudia Trenkwalder, Bastiaan R. Bloem, Rick C. Helmich

**Affiliations:** a Neurology Department, Radboud University Medical Centre, Donders Institute for Brain, Cognition and Behaviour, Centre of Expertise for Parkinson and Movement Disorders, Nijmegen, The Netherlands; b Centre for Cognitive Neuroimaging, Donders Institute for Brain, Cognition and Behaviour, Radboud University, Nijmegen, The Netherlands; c Paracelsus-Elena Klinik, Kassel, Germany; d Department of Neurosurgery, University Medical Center Goettingen, Goettingen, Germany

## Abstract

We discuss two people with Parkinson’s disease (PD), in whom tremor manifested directly following a severely stressful event. Both were initially misdiagnosed with a functional neurological disorder. These stories highlight that stress can trigger the onset of clinical manifestations of PD, by unveiling an underlying disease that had been unfolding for many years. Thus, the sudden symptom onset after a stressful event is not unique to functional disorders, and may lead to avoidable feelings of guilt if people wrongly attribute PD to this event. It remains unclear what mechanism explains this phenomenon, and why symptoms persist after the stressful event has passed.

## INTRODUCTION

“The last straw that breaks the camel’s back” is an English proverb illustrating how a seemingly minor event can trigger an unpredictably large effect, due to the cumulative effect of small preceding events. Here, we describe two individuals in whom Parkinson’s disease (PD) symptoms manifested directly following severe psychological stress. Here, stress refers to a process where environmental demands (stressors) exceed an individual’s adaptive capacity, eliciting both psychological and biological responses (the stress-response) [1]. Stressors can manifest as either physical or psychological challenges, including mentally demanding situations. Since the pathological changes associated with PD, like nigrostriatal dopamine cell loss, start many years before the appearance of the classical motor symptoms [2], we argue that severe psychological stress in these individuals was the last “straw” that caused conversion from preclinical to clinical PD.

## INDIVIDUAL 1

A now 78-year-old woman experienced a resting tremor in her right hand and foot in 2011 (at age 67), which started the day after her husband experienced a severe heart attack during a holiday (Video 1). There were no motor or non-motor symptoms before this event that could be attributed to PD. Upon returning home, after her husband’s recovery, the tremor persisted. In 2012, a neurologist performed a DAT-SPECT (dopamine transporter single-photon emission computed tomography) scan revealing left-sided nigrostriatal dopaminergic cell loss. However, since her complaints did not progress further, and given the acute onset, a functional neurological disorder was considered, and no definite diagnosis was made. Over the subsequent two years, the tremor increased and she developed motor slowness and anxiety. In 2018, she sought consultation at our university medical center, where PD (Hoehn and Yahr 2.5) was diagnosed based on marked bradykinesia, rigidity, and a typical PD resting tremor, more pronounced on the right side. Levodopa reduced her symptoms but led to motor fluctuations three years later. In 2023, she received bilateral deep brain stimulation (DBS) in the subthalamic nucleus (STN). Throughout the years, stress continued to have a significant impact on her symptoms. Interestingly, she reported symptom relief during periods of *flow*, characterized by heightened focus, particularly when translating texts (her profession).

**Figure:**
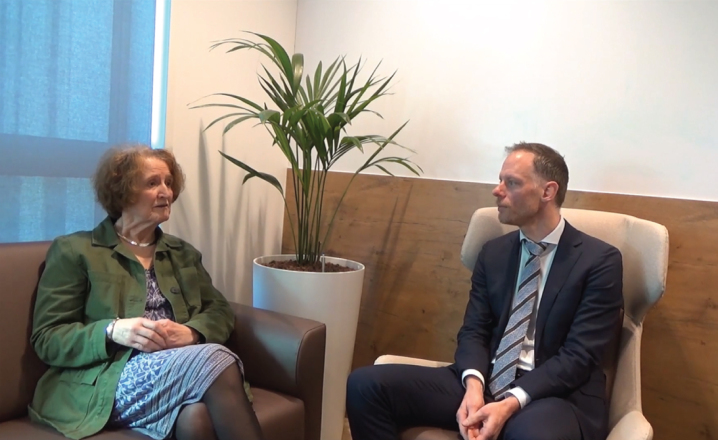
Video 1.

## INDIVIDUAL 2

In 2000, a then 50-year-old woman experienced bilateral resting tremor, more pronounced on the left side. The tremor began while standing at the open grave of her grandmother five years earlier and was initially interpreted as a psychological shock (Video 2). Despite psychotherapy, the tremor never disappeared and her condition worsened, leading to akinetic episodes and wheelchair dependency over a period of five years. Upon re-evaluation in 2005, “de novo” PD was diagnosed (at age 55), with bilateral resting tremor, akinesia and weight loss, but no cognitive decline or non-motor symptoms. Dopaminergic treatment resulted in a significant improvement, yet the tremor persisted. Motor fluctuations resulted in multiple admissions from 2009 onwards, progressing to severe PD (Hoehn and Yahr 5). In 2014, STN-DBS was performed.

**Figure:**
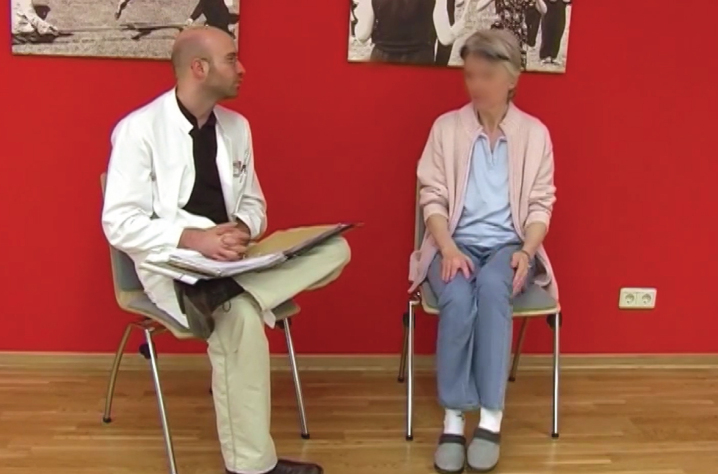
Video 2.

## DISCUSSION

We describe two people with PD, where the first clinical manifestation (tremor) was triggered by an *acute* stressful event. It is a well-known phenomenon that stress can trigger and worsen tremor [3], and that stress-reducing interventions such as mindfulness can reduce tremor [4], but the onset of tremor or other parkinsonian signs following a stressful event in previously healthy individuals is not well appreciated and warrants more attention. Currently, reports addressing similar cases remain scarce [5]. It is remarkable that in both patients, tremor was the symptoms that “unmasked” PD. This fits with previous findings showing that tremor is more subjective to stress than many other PD symptoms [6]. The mechanisms linking stress and tremor are complex and multifaceted, but a potential underlying reason is that the noradrenergic system, which is activated during stress, is thought to influence the cerebello-thalamo-cortical tremor circuitry [3, 7, 8].

In both individuals, tremor was initially considered to be functional (i.e. to have a psychological cause), given the acute onset and the association with a stressful event [9]. This led to a marked delay in reaching the correct diagnosis and in starting appropriate treatment. This initial confusion is understandable, since 70% of functional movement disorders have an acute onset, and given its high comorbidity (52%) with anxiety [9]. However, functional tremor and PD tremor have clear differences that can be objectified during neurological examination. According to current guidelines, a functional neurological disorder is diagnosed based on the presence of internal inconsistencies during neurological examination [9]. Examples of these inconsistencies are disappearance of tremor during cognitive or motor co-activation (distractibility), or a change in tremor frequency when the patient makes a voluntary movement with another limb (entrainment). Furthermore, a brief cessation of the tremor during a ballistic movement of the contralateral limb (pointing test) is a sign of a functional tremor [10]. In contrast, PD tremor often increases during motor and cognitive coactivation, and its frequency remains stable across activation conditions [11, 12]. We recommend that the diagnosis of functional neurological disorder versus PD tremor is made based on objective clinical signs, keeping in mind that functional movement disorders and PD may coexist in the same patients [10, 13]. An important learning point is that an acute onset of symptoms after a stressful event is not unique to functional disorders, but also happens in PD. In fact, this may occur regularly, because newly diagnosed people with PD commonly indicate that their symptoms emerged shortly after a stressful event [14]. Such a subacute onset is unexpected, because PD motor symptoms typically develop gradually, with slow progression for the majority of patients [15].

Another lesson is that patients and their caregivers should be educated about the link between stress and PD. The husband of the first individual experienced guilt (Supplementary Video), because he assumed that his heart attack caused her symptoms. As PD starts long before the clinical manifestation of symptoms [2, 16], a causal link with the preceding stressor is unlikely. In our experience, such feelings of guilt, specifically in relation to stressful times, are a common phenomenon. We strongly recommend that clinicians proactively ask affected individuals about a possible relation with preceding stressors, and about possible feelings of guilt, to correct wrong cognitive attributions such as: “the stressful event caused PD and therefore I am to blame for my (or my loved one’s) disease”.

The exact mechanisms by which stress can trigger the onset of PD are unknown, but a similar phenomenon has been observed in animal models. Specifically, in rats with dopamine-depleting brain lesions, severe stress “unmasked” PD-like motor symptoms (hypokinesia and rigidity), probably because the low dopamine reserves were further depleted below the clinical threshold [17]. In humans, retrospective studies report a higher PD incidence after *long-term* stress, for example in war prisoners and in people with a history of depressive episodes [18, 19]. Other studies have shown that the risk for PD increased with the number of stressful life events [20, 21]. Although the contribution of factors unrelated to stress cannot be fully excluded, this suggests a pathophysiological relationship between stress-related symptoms and PD. Possible mechanisms include neuroinflammation, oxidative stress, and altered neural connectivity, which may exacerbate neurodegeneration [22]. Furthermore, compensatory adaptations, or protective mechanisms, outside the dopaminergic system may conceivably delay symptom onset [23]. Inter-individual differences in the effectiveness of these adaptations may explain the variable duration of the preclinical phase of PD [24]. Under severe stress, it is conceivable that these compensatory changes become insufficient (due to increased demands), or stress may actively interfere with compensatory mechanisms. Noteworthy, severe stress is not the only known trigger for PD: infections, e.g. COVID-19, have also been described to immediately precede PD [25]. Similarly, the authors encountered people who developed PD shortly after undergoing surgery or anesthesia, although literature on this phenomenon is currently limited to studies examining the association between anesthesia and the subsequent risk of developing PD later in life, rather than exploring acute onsets of PD [26, 27]. This hints at mechanisms that occur both during infections and psychological stress, such as increased inflammatory tone [28]. To unravel these mechanisms, systematic longitudinal studies focusing on healthy populations are needed, such as those with idiopathic REM-Sleep Behavioral Disorder or mutation carriers at risk for PD. An assessment of life events in relation to PD symptom severity within these cohorts could provide valuable insights, aiding our understanding of the temporal dynamics of symptom onset.

A fascinating question is why PD symptoms arising after these stressful events did not disappear after the event had passed. This suggests that irreversible changes have occurred. It would be highly relevant to learn what these changes entail.

## ETHICS STATEMENT

The two individuals described in this manuscript and the husband of the first individual provided written informed consent for the usage of clinical information and video material. Approval by a medical ethics committee was not required for this case report.

## Supplementary Material

Supplementary Video 1

Supplementary Video 2

Supplementary Video 3
